# Modeling the infrared cascade spectra of small PAHs: the 11.2 μm band

**DOI:** 10.1007/s00214-021-02807-z

**Published:** 2021-08-13

**Authors:** Cameron J. Mackie, Alessandra Candian, Timothy J. Lee, Alexander G. G. M. Tielens

**Affiliations:** 1 Kenneth S. Pitzer Center for Theoretical Chemistry Department of Chemistry, University of California, Berkeley, CA 94720 USA; 2 Lawrence Berkeley National Laboratory, Chemical Sciences Division, Berkeley, CA 94720 USA; 3 van ’t Hoff Institute for Molecular Science, University of Amsterdam, P.O. Box 94157, 1090 GD Amsterdam, The Netherlands; 4 NASA Ames Research Center, Moffett Field, CA 94035-1000 USA; 5 Leiden Observatory, Leiden University, P.O. Box 9513, 2300 RA Leiden, The Netherlands; 6 Astronomy Department, University of Maryland, College Park, MD 20742 USA

## Abstract

The profile of the 11.2 μm feature of the infrared (IR) cascade emission spectra of polycyclic aromatic hydrocarbon (PAH) molecules is investigated using a vibrational anharmonic method. Several factors are found to affect the profile including: the energy of the initially absorbed ultraviolet (UV) photon, the density of vibrational states, the anharmonic nature of the vibrational modes, the relative intensities of the vibrational modes, the rotational temperature of the molecule, and blending with nearby features. Each of these factors is explored independently and influence either the red or blue wing of the 11.2 μm feature. The majority impact solely the red wing, with the only factor altering the blue wing being the rotational temperature.

## Introduction

Polycyclic aromatic hydrocarbons (PAHs) are believed to be ubiquitous in the interstellar medium (ISM) [1]. The formerly named unidentified infrared bands (UIBs), now designated the aromatic infrared bands (AIBs), are generally believed to be due to the infrared (IR) emissions of transitions between the vibrational modes of the PAHs [2, 3].

The excitation process and the following emission of these IR photons are not in thermal equilibrium [4]; a typical PAH molecule in the ISM will absorb only one ultraviolet (UV) photon per year. After absorption, the PAH becomes excited electronically and within a few hundred femtoseconds it will return to the electronic ground state where the excess energy is converted into vibrational mode excitation through a non-adiabatic process [5]. These vibrationally excited PAHs then relax largely through cooling slowly (hundreds of milliseconds) through the emission of individual IR photons. Since vibrational modes are coupled through anharmonic interactions, the temperature-dependent population of each vibrational mode perturbs the energy (i.e., emission frequency) of all other modes. During this emission process, the vibrational temperature changes and so does the shift in the frequency of the emitted photon due to the couplings. Also, as the PAH continues to emit, the amount of frequency shifting decreases. This results in what is referred to as a cascade IR spectrum, whose profile is asymmetric in shape, usually with a long red wing and little-to-no blue wing [6]. This asymmetric profile is linked to the size of the PAH, i.e., the density of states, and the initial energy of the UV photon absorbed. For a detailed review of the properties and importance of astronomical PAHs see Reference [1].

The observed IR features due to interstellar PAHs are now routinely characterized through the fitting of emission model based on theoretical IR spectra of PAHs collected in the NASA Ames PAHdb [7]. Typically, the calculations of the IR spectra are performed with density function theory methods (B3LYP being to most common functional), using low-to-modest sized basis sets (4–31G to 6-31G*), within the double harmonic approximation [8]. The profiles/bandwidths are then generated through convolving the “stick–spectra” with Gaussian and/or Lorentzian profiles (typically a standard HWHM of 15 cm$^{-1}$). These harmonic models only account for temperature-dependent populations through changes in intensity during the emission process [9]. The IR emissions of PAHs containing up to 384 carbon atoms have been calculated in such a manner [7].

The major drawback to these models is that they are not considering the dependence of the emitted IR photon frequency on the actual vibrational temperature during the cascade. This is influenced by the coupling among vibrational modes and requires an anharmonic treatment [10].

Early studies on the effect of anharmonicity on peak shifts and profiles variations of emission features [11–13] relied on temperature-dependent absorption measurements for few small PAHs in thermodynamic equilibrium [14, 15]. However, it is unclear how these measurements relate to the intrinsic vibrational properties of the PAHs and how they can be extended to larger PAHs [16]. More recently, the effects of anharmonicity on the emission profile of highly excited PAHs have also been studied using both quantum chemistry [10, 17, 18] and molecular dynamics [19, 20], or a combination of the two [21].

Nowadays, anharmonic effects can be accounted for computationally using the vibrational self-consistent field method [22], for example, or through vibrational second-order perturbation treatment (VPT2) [23, 24]. VPT2 in particular has shown great success in reproducing the experimental spectra of isolated, cold, gas-phase IR spectra of PAHs [25–31]. Anharmonic treatment of a vibrational spectrum is computationally expensive, which limits the size of computable PAHs down to only $\sim$30 carbon atoms, which is smaller than the commonly accepted size of an interstellar PAH (50–100 carbon atoms) [32]. Nevertheless, the analysis of the anharmonic IR cascades of small PAHs still provides invaluable insights into how a typical PAH emits IR photons.

Of particular interest to astronomers is the PAH features at 11.2 $\mu$m (892 cm$^{-1}$) which is attributed to out-of-plane C-H bending modes of “solo” hydrogen atoms in neutral PAHs [9, 33]. These features appear together with a satellite one at 11.0 $\mu$m (909 cm$^{-1}$), which has been attributed to both out-of-plane C–H bending modes in cationic PAHs [9], and SiPAH$^{+}$ complexes [34]. The 11.2 $\mu$m feature is characterized as having a steep blue edge, with a long red tail and the profile shows small variations which are dependent on the local environment of the observations [9, 35]. The specific shape and its variations have been explained as due to anharmonicity and to the superposition of the emission by populations of PAHs [12, 13, 36, 37]. The 11.2 $\mu$m feature dominates most of the AIB spectra of many astronomical objects, and it has been used to classify the PAHs in numerous ways, including determining their size and charge status through the 3.3/11.2 $\mu$m and 6.2/11.2 $\mu$m intensity ratios [32, 38]. A more solid understanding of the spectral behavior of the 11.2 $\mu$m feature and the influence of anharmonicity would thus be highly beneficial.

Building upon the work of Refs [17, 18], and based upon the detailed formalism provided in Ref [10], a detailed theoretical cascade model for the 11.2 $\mu$m feature is examined. The nature of this profile is explored in the context of PAH size, initial IR cascade temperatures, rotational temperature effects, and blending with neighboring features.

## Theory and background

The details of the theoretical anharmonic IR methods are explained elsewhere [25, 39, 40]. In brief, a second-order vibrational perturbation approach is used, whereby the energy of a given vibrational level is given by

1 $$\begin{aligned} \begin{aligned} E(\nu )&= \sum _k \omega _k \left( n_k + \frac{1}{2}\right) + \sum _{k\le l} \chi _{kl}\left( n_k + \frac{1}{2}\right) \times \left( n_l + \frac{1}{2}\right) \end{aligned} \end{aligned}$$

where the $\omega _k$ is the harmonic frequency, $n_k$ are the number of quanta in the $k^{th}$ vibrational mode, and $\chi _{kl}$ are anharmonic constants (see Reference [41] for their derivation).

Transition energies are then obtained by subtracting the energy of the starting level from the energy of the ending level. The transition energy from $n_{k}$ $\rightarrow$ $n_{k}-1$ for a given k$^{th}$ vibrational level is then given by

2 $$\begin{aligned} \Delta E^{\prime (k)}(\{n\}) = \omega _k + 2\chi _{kk}(n_k) + \frac{1}{2}\sum _{i \ne k} \chi _{ik} + \sum _{i \ne k} \chi _{ik}n_i \end{aligned}$$

For a detailed background of the cascade spectra of PAHs see References [10, 17]. In brief, a Wang–Landau walk [42] is performed over a given energy range for the PAHs accumulating a count of states visited in order to construct an estimate for the anharmonic density of states (DOS). After construction of the DOS, a second Wang–Landau walk is performed using the DOS as the weighting in the walk, and however, in this walk, an energy-dependent spectrum is accumulated at every energy visited. These energy-dependent spectra are then used in a cascade process. Starting at a given energy (ranging from 6 eV for a typical reflection nebula, to 10 eV for a typical HII region), the corresponding energy-dependent spectrum is accessed, and an IR photon is chosen for emission based upon its Einstein A coefficient, which in turn is proportional to the intensity (in km/mol) at a given frequency multiplied by the frequency squared. A histogram is updated with the frequency of the emitted photon. The total energy of the system is updated by subtracting the energy of the emitted photon, and then, the new corresponding energy-dependent spectrum is accessed, and a new IR photon is chosen for emission in the same manner. This process is repeated until the PAH has emitted all its energy; then, the PAH is excited again to the original starting energy, and the process is repeated until the desired resolution has been met. Ideally, the probability of a given starting energy is proportional to the distribution of photons from the stellar source times the UV absorption cross-section of the given PAH. In order to simplify the analysis and show trends, a single starting energy is selected for each simulation. This has the effect of slightly shortening the extent of red wings (due to not sampling higher starting energies), and un-biasing toward absorption at a particular energy.

A rotational temperature profile model was adapted from Reference [43]. The rotational population of interstellar PAHs is largely set by the ro-vibrational cascade. In this cascade, $\Delta J=+1$ transitions are favored by the slightly larger Einstein A coefficients, but this is counteracted by the slightly larger statistical weight of $\Delta J=-1$ transitions [44, 45]. This will lead to a Gaussian distribution characterized by an effective rotational excitation temperature. During the cascade process, each selected IR photon was subjected to an uncertainty in emission energy equivalent to a Gaussian profile generated by the rotational energy of the PAH, with a half-width–half-maximum (HWHM) estimated by the P–Q–R rotational branch separation given in Reference [43] by

3 $$\begin{aligned} \Delta \nu = 4B\sqrt{\frac{k_B T_{rot}}{hcB}} \end{aligned}$$

with *B* being the average rotational constant of the PAH in wavenumbers, $k_B$ is the Boltzmann constant, *h* is Planck’s constant, *c* is the speed of light, and $T_{rot}$ characterizes the average rotational population distribution that is set by the cumulative effect of the vibrational cascades

4 $$\begin{aligned} \begin{aligned} T_{rot}&= h c B J_{IR}^2 \\&= \frac{ h c \nu _{mean}}{6 k_B} \end{aligned} \end{aligned}$$

where $J_{IR}$ is the most probable rotational quantum number, and $\nu _{mean}$ is the average vibrational frequency of the emitting vibrational modes of the PAH. The factor 6 in this expression reflects the summation over the rotational K ladders. Radiative and collisional deexcitation of the rotational states will drive the actual rotational excitation temperature of the molecule down from this value, but this effect is small for the interstellar emission regions [43]. While the exact rotational constants can be used in this model, for the analysis, in sect. 4.5, the following approximation for the rotational constants is used

5 $$\begin{aligned} B \approx 2\times 10^{-3}\left( \frac{50}{N_c}\right) ^2 \text {cm}^{-1} \end{aligned}$$

where $N_c$ is the number of carbon atoms in the PAH. This allows for a systematic — but approximate — approach to the rotational broadening by varying the effective “size” of the PAH ($N_c$)

## Methods

This work follows largely the methods and parameters outlined in previous work [10, 17] for generating the IR cascade spectra. The geometry optimizations, harmonic IR calculations, as well as the calculation of the quartic force field (QFF) terms, were performed using the Gaussian16 software package [46]. The VPT2 treatment was performed using a locally modified version of the SPECTRO [47] for direct control over resonances and polyad sizes). All calculations were performed using density functional theory (DFT), using the B3LYP [48, 49]/N07D [50] (a basis set based on 6–31G, with a limited number of diffuse and polarized functions, shown to perform well for anharmonic calculations.) functional/basis set combination, with the convergence parameters recommended for anharmonic calculations [27]. Comparison of the theoretical anharmonic zero-Kelvin spectra with measured ion-dip spectra for a variety of PAHs shows excellent agreement (to within 0.3%) in peak frequency without the need for correction factors [28–31].

Anthracene (C$_{14}$H$_{10}$) — a molecule with a strong solo out-of-plane bending mode — is used as a stand-in for the analysis of these parameters. These trends are indicative for the behavior of the out-of-plane bending modes of all neutral PAHs with strong 11.2 $\mu$m features. However, the data set is currently too limited (in both number of PAHs, and size of PAHs) to draw any firm size-dependent extrapolations at this point.

## Results and discussion

The profile of the 11.2 $\mu$m feature is found to be controlled mainly by three variables: the energy of the absorbed UV photon before the cascade process, the size of the emitting PAH, and the rotational temperature of the PAH (which also has a size dependence). However, the variation in profiles due to size is found to manifest in multiple ways including: changes in the DOS, changes in the anharmonic character, and changes in the relative intensities of the other vibrational modes. In addition to these intrinsic variations, the 11.2 $\mu$m profiles can also be affected through external factors, such as blending with neighboring features. Due to the complicated nature of these intertwined effects, variations in each aspect (cascade energy, rotational temperature, DOS, anharmonic character, relative intensity, and blending) are considered individually.

**Fig. 1:**
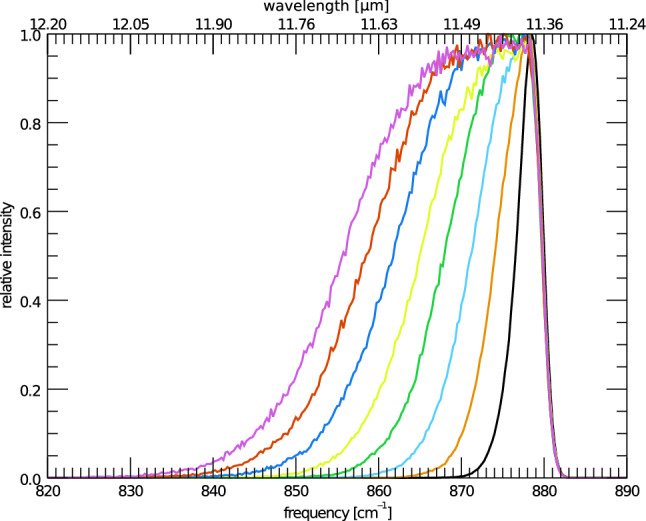
The IR cascade spectra of the isolated 11.2 $\mu$m feature of anthracene beginning from varying maxima of energy absorbed. Purple (left most) is a maximum of 8 eV absorbed, black (right most) is a maximum of 1 eV absorbed, with the remaining spectra being step sizes of 1 eV in between the two extremes

### Cascade energy

The first variable, the maximum energy absorbed, is the easiest to model and explain. Figure 1 shows the IR cascade spectrum of anthracene at various starting cascade energies, with the other variables being kept constant (3.3 to 11.2 $\mu$m ratio set to zero (see sect. 4.4), and rotational broadening set to a HWHM of 1 cm$^{-1}$ (see sect. 4.5)). As can be seen, the extent of the red wing is controlled directly by the cascade starting energy, and the blue wing is left unchanged. Additionally, no change in peak position is observed. The growth of a red wing, and lack of change in blue wing and peak position can be explained by the anharmonic nature of the 11.2 $\mu$m feature. All significant anharmonic couplings between the solo CH–modes and all other modes are found mainly to lower the energy of the vibrational mode. That is to say, the anharmonic constants used in Eq. 2 are found to be largely negative [10]. This leads to a broad emission band at lower frequencies when the PAH has a high internal energy, and a sharply peaked emission band at low internal energies, which in turn does not overshoot the zero-Kelvin peak position (879.7 cm$^{-1}$).

**Fig. 2:**
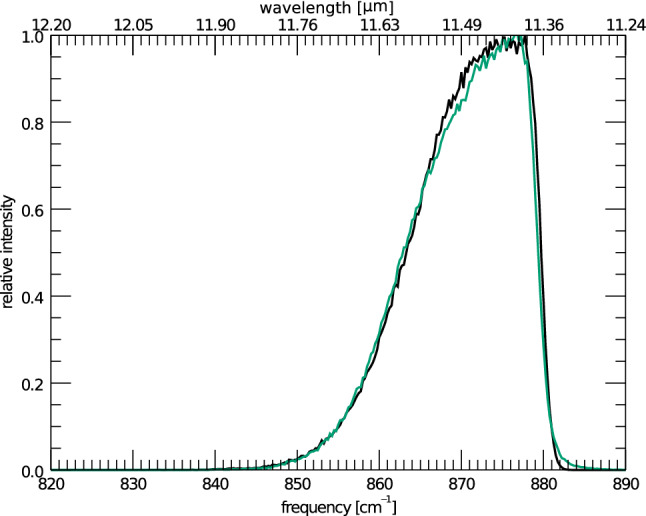
The IR cascade spectrum of the isolated 11.2 $\mu$m feature of anthracene starting from a maximum of 6 eV absorbed (green) compared to the same feature of tetracene starting from a maximum of 8 eV absorbed (black) (the band position of tetracene has been shifted by 21.5 cm$^{-1}$ to align with anthracene)

### Density of states

Figure 2 shows the cascade spectrum of anthracene (C$_{14}$H$_{10}$) at 6 eV, with the equivalent cascade spectrum of tetracene (C$_{18}$H$_{12}$) at 8 eV. The two profiles are almost identical.

The DOS of a given PAH is related directly to the number of vibrational modes of the PAH. As the size of PAHs is increased, so too does the DOS. The similarity in molecular structure and vibrational frequencies of PAHs results in a density of states that has great resemblance for all PAHs. As a result, to a good approximation, the microcanonical vibrational excitation temperature scales inversely with the number of vibrational modes to some power (typically 0.4) [43]. This translates into narrower cascade features for larger PAHs for a fixed excitation energy relative to that for a smaller PAH molecule for the same excitation energy. This means, all else being equal, a larger PAH will produce a similar cascade profile as a smaller PAH that is excited by a lower energy, as shown in Fig. 2.

**Fig. 3:**
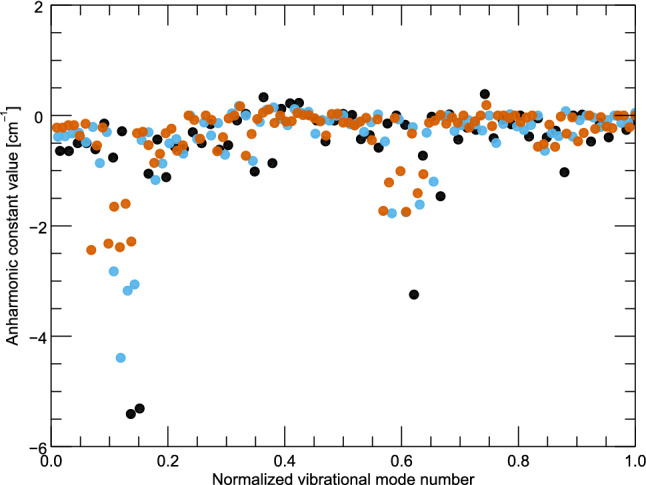
The anharmonic constants of the 11.2 solo out-of-plane $\mu$m vibrational modes of anthracene (black), tetracene (blue), and pentacene (brown). The vibrational mode numbers have been normalized such that similar couplings to vibrational modes align between the three PAHs when plotted

### Anharmonic character

In the VPT2 treatment, each vibrational mode is coupled to all other vibrational modes through their anharmonic constants. The degree by which the frequency of a particular vibrational mode $\nu _i$ is perturbed is given by Eq. 2. Figure 3 plots the anharmonic constants for the 11.2 $\mu$m feature of anthracene (C$_{14}$H$_{10}$), tetracene (C$_{18}$H$_{12}$), and pentacene (C$_{22}$H$_{14}$) as a function of normalized vibrational mode number (n/max(n)), such that the equivalent vibrational mode types align roughly. The lower numbers represent vibrational modes which are higher in energy. The value seen around the normalized mode number of 0.15 represents the coupling of the 11.2 $\mu$m vibrational mode to the CH–stretching modes ($\sim$ 3.3 $\mu$m, 3030 cm$^{-1}$), and the values seen around 0.65 are the coupling to the out-of-plane CH–bending modes ($\sim$ 11.2 $\mu$m, 892 cm$^{-1}$), including the self-interaction. As can be seen, the absolute value of the large negative individual anharmonic constants decreases with size of the PAH, however, since the number of vibrational modes increases, the total sum of the anharmonic constants remains nearly constant. These two effects largely cancel out when considering the zero-Kelvin anharmonic correction, leading to very similar shifts with respect to the harmonic position. However, the same amount of vibrational energy in a PAH results in less and less broadening as the size of the PAH increases; this is because the spectator modes — the excited vibrational modes coupled to the solo out-of-plane mode but not emitting — are coupling through smaller anharmonic constants thus causing a smaller frequency shift. This effect couples with the DOS effect mentioned in sect. 4.2 resulting in narrower features for larger PAHs.

**Fig. 4:**
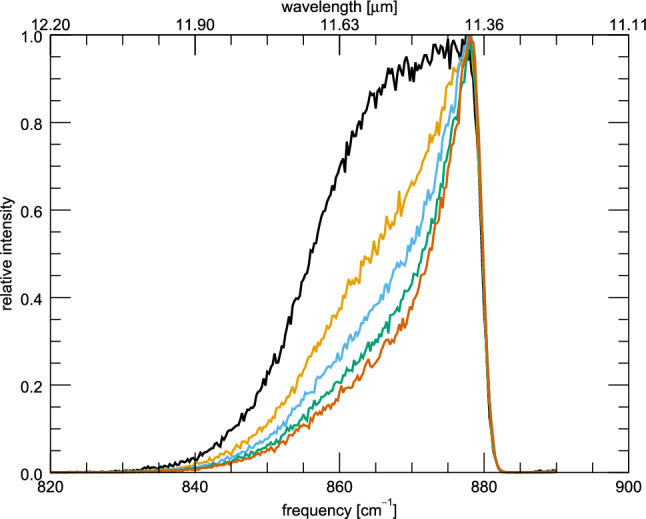
The IR cascade spectrum of the 11.2 $\mu$m feature of anthracene at 8 eV with the intensity of the 3.3 $\mu$m emission region absent (black), scaled by 0.5 (orange), unscaled (blue), scaled by 1.5 (green), and scaled by 2.0 (brown)

### Relative intensities

The resulting profile of a cascade emission band is the convolution of the emission profiles at different internal energies weighted by the fraction of the energy that is instantaneously emitted in that particular band at that internal energy. Hence, the profile will depend on the relative strength of all modes. We illustrate this here by varying the relative integrated IR strength of the C–H stretching modes relative to the CH out-of-plane bending modes. Figure 4 shows the 11.2 $\mu$m feature of anthracene where the ratio of the 3.3 to 11.2 $\mu$m band intensities has been adjusted artificially to different values (0.5, 1.0, 1.5, 2.0). As can be seen, the red wing fades away at higher 3.3 to 11.2 $\mu$m ratios. This occurs because at higher internal energies (i.e., the start of the cascade), more IR energy is being released through the 3.3 $\mu$m vibrational modes, but as the PAH cools eventually the 11.2 $\mu$m begins to dominate the emission. This has the affect of turning a convex profile into a concave profile. The 3.3 to 11.2 $\mu$m ratios have been shown previously to correlate directly with size of the PAH [32]. This means smaller PAHs will have broader profiles, similar to the black profile of Fig. 4, while larger PAHs will tend toward the slimmer brown profile.

**Fig. 5:**
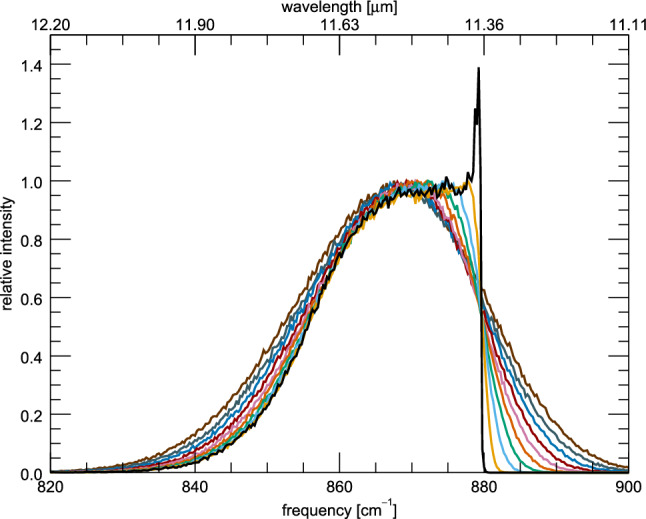
The IR cascade spectrum of the isolated 11.2 $\mu$m feature of anthracene at 8 eV with rotational temperatures ranging from 0 K (black) to 400 K (brown)

**Fig. 6:**
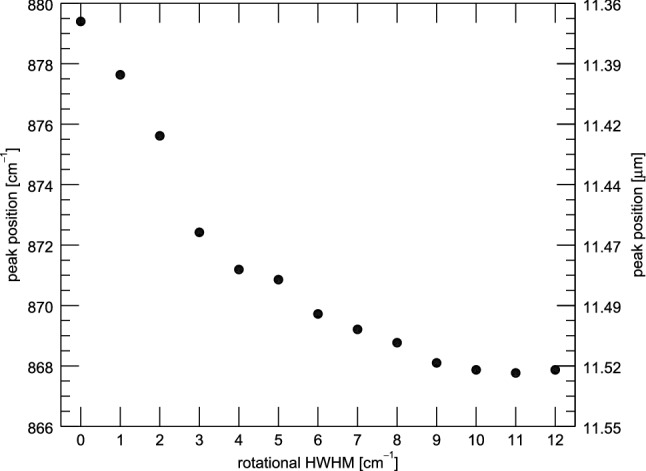
The shift in peak position of the 11.2 $\mu$m feature of an 8 eV cascade for anthracene, as a function of rotational broadening. The peak position moves toward the red and eventually stop with increased rotational broadening

### Rotational temperature

Figure 5 shows an 8 eV IR cascade spectrum of anthracene adopting different rotational broadenings through varying $N_C$ in Eq. 5, with all other parameters remaining fixed. As can be seen, the profile of the steep blue wall of the cascade feature is controlled by the rotational temperature. In fact, rotational temperature broadening is the only factor found to control the shape of the blue wing. Additionally, the sharp peak at the zero-Kelvin position can be seen to blend out at higher rotational temperatures. This portion of the profile is emitted at low internal excitation temperatures and is sharply peaked due to the lack of coupling to spectator vibrational modes at low energy. The blue rise reflects then mainly the adopted rotational broadening.

The peak position of the 11.2 $\mu$m feature is also found to be controlled mainly by rotational temperature. Starting cascade energy has no effect on the position of the 11.2 $\mu$m feature (see Fig. 1, although neighboring features may also play a role (vide infra)). Figure 6 illustrates the change in peak position with rotational temperature of anthracene. The 11.2 peak position shifts toward the red as the rotational temperature increases, eventually reaching a plateau. At low internal energies, the dominant source of broadening for the feature is due to rotational temperature. Instead, at higher internal energies, the dominant source of broadening is the vibrational temperature. This has the effect of eroding the steep blue wing of the feature up until the profile appears symmetric, and the shifting of peak position stops.

Rotational broadening is related to the size of the PAH, since the rotational constant B is inversely proportional to the size of the PAH squared (Eq. 5). Larger PAHs will therefore have a steeper blue wing than smaller PAHs at similar rotational temperatures.

**Fig. 7:**
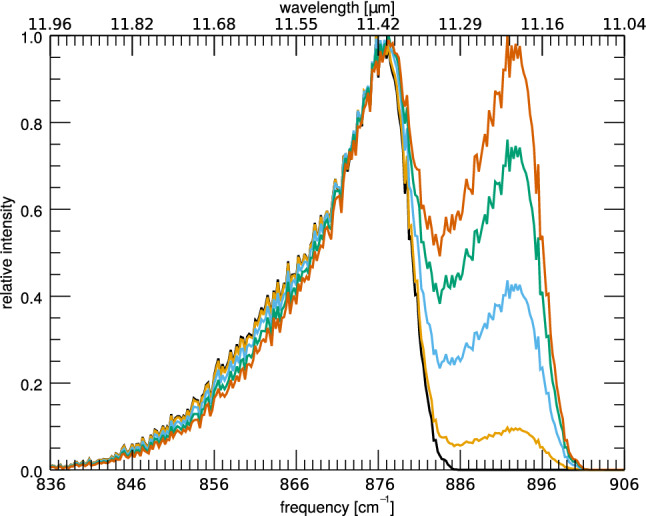
The IR cascade spectrum of the 11.2 $\mu$m feature of anthracene from a 8 eV cascade with an artificial neighbor at 894 cm$^{-1}$, mimicking the 11.0 $\mu$m feature. Black has a 11.0 to 11.2 $\mu$m intensity ratio of zero, orange is 0.1, light blue is 0.5, green is 1.0, and brown is 1.5

### Blending

Figure 7 shows the 11.2 $\mu$m cascade feature of the full IR cascade of anthracene changing through the increase in the intensity of a cascade “mock” feature at 11.0  $\mu$m. Neighboring features are also found to affect the profile of the 11.2 $\mu$m feature through blending. This blending is observed to change the peak position, blue wing, and red wing all subtly.

More interestingly, the red wing of the neighboring feature increases the apparent intensity of the 11.2 $\mu$m feature giving the illusion that the ratio of 11.2 to 11.0 intensity is not increasing as much as it is. The brown spectrum of Fig. 7, for example, has a ratio of the 11.0 to 11.2 of 1.5, but a naïve fit to the spectrum may conclude incorrectly that the 11.2 $\mu$m feature has an equal intensity to the 11.0 $\mu$m feature.

### Photo fragmentation

In this analysis, the effect of photo fragmentation on the energy cascade has been neglected . In principle, at high internal excitation, fragmentation becomes a competitive energy loss channel. As a result, molecules with such high internal energies do not contribute to the emission process and the increase in the width is truncated at these energies [17]. In practice, this is of limited interest as in interstellar regions where H–loss becomes important, these PAHs are rapidly stripped of all their H’s [51] and then destroyed.

## Conclusions

While qualitative in nature, this work provides insight into the profile of the 11.2 $\mu$m feature. In order to draw more firm conclusions, and extrapolate to larger PAHs a bigger data set would be required. Unfortunately, anharmonic calculations of PAHs are not straightforward and are hampered by some unresolved issues [27, 28, 31]. Computational power remains a roadblock for calculating anharmonic spectra of larger PAH species. However, it is not the only roadblock. Anharmonic computations of larger PAH molecules have been found to be unsuccessful, due largely to numerical instabilities, including large anharmonic corrections of several hundreds of wavenumbers in out-of-plane bending modes (where corrections are typically in the tens of wavenumbers). The cause for this is not known; it could be due to implementation issues in the DFT methods or it may be due to an unresolved issue for out-of-plane bending vibrations [52]. Additionally, experimental spectra of large PAHs under low-temperature gas-phase conditions are lacking in order to verify the anharmonic calculations themselves. Nevertheless, much can be extracted from this subset of small PAHs.

The 11.2 $\mu$m feature profile is found to be primarily controlled by the size of the PAHs. However, individual aspects are affected by PAH size in different subtle ways.

The initial cascade energy controls the extent of the red wing when considering a single species. However, the extent of the red wing at a given cascade energy is dependent on size in two ways: one, the DOS of larger PAHs spreads the energy over many more vibrational modes, resulting in a cooler cascade than a smaller PAH; two, the individual anharmonic coupling constants become smaller with increasing PAH size, resulting in smaller band shifts at all energies. Both of these effects mean a shorter red wing as the size of the PAH increases.

The shape of the red wing is controlled by size through the relative intensities of the 3.3 to 11.2 $\mu$m features, which in turn are controlled by the size of the PAHs. A large 3.3 to 11.2 $\mu$m ratio is typical of small PAHs, while a small ratio is typical of large PAHs. A small 3.3 to 11.2 $\mu$m ratio in turn results in a broad convex profile, while a large 3.3 to 11.2 $\mu$m ratio results in a concave profile.

The profile of the blue wing is controlled by the rotational temperature of the PAH, and to a lesser extent by blending with neighboring features only at higher frequencies than the 11.2 $\mu$m feature itself. Larger PAHs have smaller rotational broadening, due to smaller rotational constants, resulting in steeper blue–wings for larger PAHs [12, 13].
